# An Automated Electronic System in a Motorized Wheelchair for Telemonitoring: Mixed Methods Study Based on Internet of Things

**DOI:** 10.2196/49102

**Published:** 2023-11-08

**Authors:** Luma Carolina Câmara Gradim, André Luiz Maciel Santana, Marcelo Archanjo José, Marcelo Knörich Zuffo, Roseli de Deus Lopes

**Affiliations:** 1 Polytechnic School, Interdisciplinary Center for Interactive Technologies and Institute of Advanced Studies University of Sao Paulo São Paulo Brazil; 2 Instituto de Ensino e Pesquisa Insper São Paulo Brazil

**Keywords:** eHealth systems, telemonitoring, inertial measurement unit, IMU, sensors, Internet of Things, IoT, pressure injury, wheelchair, mobile phone

## Abstract

**Background:**

Wheelchair positioning systems can prevent postural deficits and pressure injuries. However, a more effective professional follow-up is needed to assess and monitor positioning according to the specificities and clinical conditions of each user.

**Objective:**

This study aims to present the concept of an electronic system embedded in a motorized wheelchair, based on the Internet of Things (IoT), for automated positioning as part of a study on wheelchairs and telemonitoring.

**Methods:**

We conducted a mixed methods study with a user-centered design approach, interviews with 16 wheelchair users and 66 professionals for the development of system functions, and a formative assessment of 5 participants with descriptive analysis to design system concepts.

**Results:**

We presented a new wheelchair system with hardware and software components developed based on coparticipation with singular components in an IoT architecture. In an IoT solution, the incorporation of sensors from the inertial measurement unit was crucial. These sensors were vital for offering alternative methods to monitor and control the tilt and recline functions of a wheelchair. This monitoring and control could be achieved autonomously through a smartphone app. In addition, this capability addressed the requirements of real users.

**Conclusions:**

The technologies presented in this system can benefit telemonitoring and favor real feedback, allowing quality provision of health services to wheelchair users. User-centered development favored development with specific functions to meet the real demands of users. We emphasize the importance of future studies on the correlation between diagnoses and the use of the system in a real environment to help professionals in treatment.

## Introduction

### Background

Wheelchairs are assistive technology products that provide mobility and independence. Wheelchair users who perform different occupational roles on a daily basis remain seated for long periods and carry out their activities of daily living. Therefore, they require good alignment and postural positioning to be functional and to have a good quality of life. This requires personalized prescriptions and adaptations with professional follow-up [1].

Even if there is no perfect posture, as each daily activity requires a different movement and posture, the literature shows that sitting time must be dynamic. Correct changes in positioning in the wheelchair, with a certain frequency and duration, can favor occupational functions and health of the skin that maintains contact with the support surfaces, in addition to promoting better pressure distribution on the seat close to the backrest.

No static biomechanical model can maintain the human body (in the same position) for a long time without consequences [2-4], as it can cause damage to the posture and health of wheelchair users.

Impairments in the postural alignment and health of wheelchair users can be aggravated by complications, such as pressure injuries, contractures, postural deformities, and decreased tissue blood perfusion in the gluteal region. Therefore, it is essential for wheelchair users to maintain dynamic and balanced posture during their daily routines.

Dynamic wheelchair positioning systems, such as tilting and reclining, favor pressure relief in the seat and backrest regions; this can prevent clinical complications and favor postural adaptation, biomechanics of the sitting position, breathing, and comfort [1,3,4].

Tilt operates in the sagittal plane, where there is a change in the orientation of the wheelchair seat angle relative to the ground, maintaining the angle between the seat and backrest. Recline provides a change in the orientation of the backrest, maintaining a constant angle of inclination in relation to the ground and increasing the angle of inclination between the seat and backrest [1,5].

Several studies on the subject of angles and changes in positioning in a wheelchair have shown that tilting and reclining positions, especially at greater angles, have a reduced risk of developing postural contractures and pressure injuries. In addition, it can increase the person’s comfort and offer better postural stability [1,5,6]. However, each case must be analyzed individually by a professional specialist.

There are different methods for performing manual changes in positioning, such as push-ups, using upper limb techniques. However, when there is any compromised component in the upper limb, spasticity, reduced mobility, deformities, and joint or postural limitations that may limit the user’s performance for independent position changes, the use of automated dynamic wheelchair positioning system wheels may be a viable option [4,5].

Therefore, tilting and reclining maneuvers applied correctly, with adequate professional follow-up and individualized evaluation focusing on occupational activities to favor functionality, bring more quality of life and health to wheelchair users [3,4,6].

### Monitoring of a Wheelchair Use Function and Technology Interaction Gap

The use of a wheelchair as an assistive technology device and its functions, recommended and monitored by health professionals, offers the possibility of promoting mobility, functionality, quality of life, and independence of users on a daily basis.

However, health care and rehabilitation services for wheelchair users often do not understand what happens in other contexts outside the care environment, as well as in patients’ environmental factors, which makes monitoring and adequate therapeutic treatment difficult, generating delays and slowness in the rehabilitation process with decreased quality [7,8].

Researchers in the field [9] show that documented clinical practices with wheelchair users reveal little or no time dedicated to training the positioning functions of this assistive technology. In these circumstances, the lack of professional monitoring of the functional use of assistive technology can lead to a decrease in daily use, abandonment of technology, and an increased need for help. In addition, users are also concerned about the high investment in assistive technology equipment such as wheelchairs [9,10].

Therefore, in addition to having a multidisciplinary and specialized team, rehabilitation services also seek growth in improvements, facilities, and accessibility through computerized, information, and technological systems [11].

In the context of monitoring changes in the position of wheelchair users, the literature shows the possibility of indicating changes in daily positioning with technologies in their favor, such as sensors, pressure relief, and comfort at small angles (≤15°), which also favors occupational involvement [12].

However, although all inclined angles tended to favor the position and health of wheelchair users, not all of them favor occupational performance. The impact of repositioning the human body at greater angles in a motorized wheelchair leads to dynamic changes in the center of gravity, field of vision, and manual reach, which directly affect the mobility and occupational performance of the user.

Furthermore, the performance of the wheelchair is a relevant factor in operating and changing positions. Therefore, when carrying out this development study of an automated electronic positioning system for wheelchairs, we also considered, in addition to health promotion, the variability in the position of the center of gravity of the human body caused by the inclination of the seat in different soils, which translates into static and dynamic stability and, therefore, operational safety.

Considering the individuality of each case and the importance of personalizing therapeutic care with the possibility of monitoring by a professional, this study aimed to develop an electronic system embedded in a motorized wheelchair for automated positions prescribed individually by a health professional and capable of telemonitoring the positions.

In an interdisciplinary context, our study also offers a proposal for the interactive development and formative evaluation of an innovative technological solution based on the real problems of wheelchair users. It involves a team composed of occupational therapists, electrical engineers, computer engineers, and mechanical engineers.

This study is part of a larger research project developed by the Support Program for Graduate Studies, Scientific and Technological Research in Assistive Technology in Brazil (number 59/2014), with a funding line for innovation in assistive technologies and studies on disability.

### Literature Review

José and de Deus Lopes [13] developed the Power Wheelchair Open Platform. They presented the architecture, detailing hardware and software, to collaborate with the Open Hardware community and provide an economic platform for research purposes. It is an electronics project for motorized wheelchairs, containing an advanced modular structure with a Bluetooth connection, controller ports (for the main user and the caregiver), and infrared emissions.

In 2016, Martinazzo et al [14] continued the Power Wheelchair Open Platform project, developing the Motion Assistant, a Bluetooth-enabled motorized wheelchair control module, based on industry demands and considering the International Organization for Standardization (ISO) 7176 standard series (which defines requirements suitable for industrial manufacturing with test methods for manual and powered wheelchairs).

Gradim et al [15] conducted a review of publications on the functions of wheelchair positioning—tilt and recline. This study was essential to understand the challenges, limitations, and demands of the theme, as well as the state of the art, to deepen the development of an innovative electronic system for positioning functions in motorized wheelchairs. In addition, an experimental study was conducted involving people with spinal cord injuries and users of motorized wheelchairs to understand the health and disease processes as well as complications such as pressure injuries, which occur because of the lack of changes in wheelchair positions.

In 2020, Gradim et al [16] conducted a systematic review of the literature on Internet of Things (IoT) services and applications in rehabilitation. This study aimed to strengthen the links between therapies with wheelchair users and the technologies developed in this area. However, the focus on IoT is a growing trend in research involving the development of technological solutions with sensor applications in several fields (eg, health, education, industry, and entertainment).

Campeau-Vallerand et al [9] presented relevant topics for the construction of an electronic system focused on the tilt function in motorized wheelchairs, such as the ability to use the physical components of a system interface, feedback options on specific tilt parameters, and the interaction between the user and clinicians, which are positive factors for the implementation of a technology. This study was evaluated and validated by clinicians and stakeholders, focusing mainly on the evaluation aspect of interactions with users. In addition, the authors presented hardware with 2 accelerometers (InvenSense [InvenSense Inc] and MPU-6050 [TDK InvenSense]) attached to the wheelchair, both at the power base and backrest, to measure the difference between the inclination angles in relation to gravity. Compared with the proposed study, the CONTAV (cadeira de rodas motorizada com CONTrole AVançado) hardware has cloud IoT technology that controls up to 3 motorized wheelchair actuators, inhibits sensors for each seat function, and monitors the seat angle with the sensor module MPU-6050 inertial measurement unit (IMU), which has a 3-axis gyroscope sensor and a 3-axis accelerometer on the same chip and microelectromechanical systems.

Wu et al [17] developed and evaluated a mobile device app to encourage repositioning behaviors based on professional recommendations, with personalized reminders for the user. The developed product was compatible with only 1 manufacturer, reaching a small number of users and professionals. In addition, the authors presented limitations in the performance of the usability test after presenting a functional prototype to the final user. Furthermore, they had some complications with the use of accelerometers in moving wheelchairs and did not use wireless data transmission. However, it has made important contributions to the technological development in this area.

Comparing the app developed by Wu et al [17] with the system proposed in this study, a collaborative design study was developed from the first moment of the system design, use of accelerometers and other devices with wireless transmission, IoT technology with sensors, and IMU in an automation system.

Several studies have been published with the aim of understanding the actions in research at the intersection between the areas of technological systems and changes in tilt and recline positions for the prevention of pressure injuries, for postural health, and for functionality in a wheelchair. The strands of solutions found are somehow correlated with the technological solution proposed in this study, as we seek to list the most favorable results and methodologies for building the system presented in this study [6,9,12,17].

All the studies cited in this section are references for the design of the system that will be described in this study, as they present proposals for technological, interdisciplinary, and user-centered research, which favors the applicability of a solution in practice. This study also includes research with users and, in addition, proposes a new IoT technology with specific functions and indicators systematized in a system for health professionals who prescribe wheelchairs and perform treatments with users. The system provides a treatment guide for professionals with recommendations, guidelines, and objectives for an intervention plan that must be carried out in a service with wheelchair users. It also favors the follow-up of the intervention plan with real feedback and telemonitoring of the follow-up of the treatment plan for wheelchair users, following systematized health indicators recommended by the World Health Organization and public policies of the Unified Health System from Brazil. Furthermore, the developed system is embedded in a wheelchair with the capability of remote management by the professional and self-management by the wheelchair user using smart mobile devices.

On the basis of the exposed content, there are still doubts about ways to help prevent pressure injuries in wheelchair users, aiming at self-management; self-maintenance of health care; and changing angles, frequency, and duration.

This study proposes an architecture with component concepts (ie, hardware and software) of an electronic seat system that performs programmable tilt and recline in a wheelchair. The influence on the biomechanical parameters and usability of the technology, adaptable to the daily context of wheelchair users, were considered. In addition, it is recommended that the system be easily transported and inserted into the user’s daily life to prevent pressure on the seat.

Therefore, it is important to consider the feasibility hypothesis of a technical electronic system that helps prevent the pressure exerted on the seat, both for users and for technological devices, with functions based on a specialized intervention plan by a health professional.

### Objectives

The purpose of this study was to present concepts for the development of a monitoring system for the use of the power tilt and recline architecture of the wheelchair and CONTAV hardware (motorized wheelchair with advanced control), which is an IoT system with IMU sensors in an automation system for tilting and reclining positions in a motorized wheelchair, with the capacity for professional electronic prescription and telemonitoring. In addition, we developed a professional interface with a survey of specific functions for usability in the treatment and monitoring of wheelchair users.

## Methods

### Participants

To survey development demands (co-design process), 3 groups participated in the study: the first group comprised 16 wheelchair users, the second group comprised 57 health professionals (31 occupational therapists and 26 physiotherapists) with experience in prescribing motorized wheelchairs with motorized seating functions, and the third group comprised 9 professional specialists in technology.

The inclusion criteria for participation in the research were as follows: group 1 participants needed to be wheelchair users, group 2 participants were required to have professional experience working with wheelchair users, and group 3 participants were expected to have experience in the technological development of health care systems.

Of the 57 health professionals, 5 (9%) were chosen by a convenience sample to participate in the iterative development of the system and usability tests in this study [17].

All participants had to have access to a smartphone to monitor the development and improve system functions during the iterative development, in addition to the notions of opening an app, using a cell phone and app tools.

As exclusion criteria, group 2 excluded those who had never had experience with wheelchair users, and group 3 excluded those who had never conducted work or research in technology in the health area.

### Ethical Considerations

The Research Ethics Committee of the University Hospital of the University of São Paulo forwarded and approved this research (CAAE:26600719.0.0000.0076).

All participants agreed to participate in the study and signed an informed consent form.

### Methodological Procedures

The methodological process of the study for developing an automated electronic system in a wheelchair for telemonitoring, following the stages of user-centered design (UCD), is summarized in 4 stages of the research, including the data collection instruments for the app view (Table 1).

Owing to the social distancing imposed by the COVID-19 pandemic, all data collection activities were conducted remotely via the Google Meet (Google) tool and software development and evaluation activities.

The first stage was based on a literature review and previous studies on the structural design of the methodological stages and development of the questionnaires. The second stage was to make the questionnaires available on the internet (Google Forms) and disseminate them to the participants. In this step, we also collected qualitative responses that provided inherent data for the base construction of prototype 1 and selected a smaller sample of 5 participants (ie, health professionals) for formative assessment of system usability and satisfaction. Furthermore, in this step, three versions of the prototype were created: (1) on paper, (2) programming using Figma (Figma, Inc) with modifications from the first version to present the usability of the system, and (3) a postmodification presentation with a general test of all the features and functions for formative evaluation is shown in Figma. The fourth stage was not carried out owing to the COVID-19 pandemic.

**Table 1:** Steps of the methodological process of the development study based on the UCD^a^ approach.

Stages	Description
First stage	Design of experiments Development of assessment protocol, including a semistructured questionnaire, specifying standardized instruments for data collection with participants, based on WHO^b^ guidelines and scientific evidence
Second stage	User experience assessment: application of semistructured questionnaires 16 wheelchair users (questionnaire 1) 57 health professionals (31 OTs^c^ and 26 physiotherapists; questionnaire 2) 9 professional specialists in technology (questionnaire 3) Definition of application of standardized evaluations (SUS^d^ and ASQ^e^) at the end of all tests Post Q analysis: development of prototype 1, paper version—UCD 1 Establishment of criteria and measures for uniform evaluation of prototypes
Third stage	Formative assessment interview: evaluating the first prototype UCD 1 pre test 5 OTs (SUS and ASQ) Analysis, adaptations, and generation of the prototype 2 (with Figma [Figma, Inc])—UCD 2 Formative assessment interview: evaluating the second prototype—UCD 2 Test Same 5 OTs (SUS and ASQ) Analysis, adjustments, and development of prototype 3—UCD 3
Fourth stage	Final assessment of third prototype, functional—UCD 3 5 wheelchair users and 5 OTs in a real situation of use (SUS, ASQ, Quebec User Evaluation of Satisfaction with Assistive Technology [QUEST] 2.0, and Post-Study System Usability Questionnaire [PSSUQ])

^a^UCD: user-centered design.

^b^WHO: World Health Organization.

^c^OT: occupational therapist.

^d^SUS: System Usability Scale.

^e^ASQ: After-Scenario Questionnaire.

### Protocol and Data Analysis

This study used methodological research techniques from the action design research method with a user-centered development approach to design an interoperable IoT-based system through a co-design process (researcher and end user) [18].

Understanding and knowledge of the system’s data content was the first step toward building the software (ie, version 1 of the paper prototype), which took place through the qualitative analysis of the answers to the questionnaires, questionnaire 1, questionnaire 2, and questionnaire 3, developed by the authors with this objective, and answered on the web by 16 wheelchair users, 57 health professionals, and 9 technology specialists, respectively.

In directive terms, the system software developed based on the methodological description above, with iterative and coparticipatory development with the research participants, was generated with functionalities in interoperability (eg, system capable of operating, functioning, or acting with another) based on IoT with the collection and sampling of data from sensors, sending and receiving data to the cloud, and therapist-patient communication. All functional aspects of the system were built according to the demands of the professionals who participated in development.

Several factors were determined during the system design phase, namely, the development of a web application, microcontroller or microcontrollers and single-board computer for IoT applications, the technology of database management systems for cloud applications, cloud platforms for viable data storage, and solutions for presenting cloud data to users (ie, both therapists and patients).

After the development of the first prototype of the system, each change in iterative development was presented to 5 research participants (convenience sample). At the end of the presentation of the prototype via a video with a demonstration of the use of hardware and software and their functions, a formative evaluation was carried out.

Two formative evaluation sessions (V1 and V2) were conducted at UCD 1 and UCD 2 stages, aimed at the acceptability and usability of users in relation to the system. These evaluations included a formative evaluation of the system by end users in the prototype development phase to identify and solve problems that could influence the user experience [9]. At the end of each presentation, the After-Scenario Questionnaire (ASQ) and System Usability Scale (SUS) were applied. Descriptive statistical analyses were performed using these quantitative instruments.

The ASQ is a reliable and valid measure of satisfaction and consists of a 3-item questionnaire to assess user satisfaction with the usability of a system. The items were ease of task completion, task execution time, and adequacy of supporting information (internet-based help and messages) on a 7-point scale (lower scores indicate greater satisfaction). The overall ASQ score was obtained by averaging the scores for the 3 items.

In addition, the SUS was applied as a usability and learning assessment measure, consisting of 10 questions separated by 2 independent data sets—usability (8 items) and learning (2 items)—on a 5-point scale.

The SUS scores ranged from 0 to 100. According to the following equation, it is calculated by multiplying the sum of the scores referring to the even and odd questions, resulting in a single representative value for the general usability of the system:

value = [(𝑃1 − 1) + (5 − 𝑃2) + ⋯ + (𝑃9 − 1) + (5 − 𝑃10)] × 2.5

where *Pi* is the score obtained for each question with *i* ranging from 1 to 10.

Suggestions and comments regarding these improvements are also provided. The materials collected qualitatively through observation and listening were analyzed by content analysis and classified by system characteristics by the interdisciplinary team. In this study, the concepts that emerged from the qualitative analysis of the data collected from the questionnaires (questionnaire 1, questionnaire 2, and questionnaire 3) and values from the quantitative analysis for the standardized assessments of SUS and ASQ, as well as the result of the design of the prototypes of step 3, will be displayed in the session results.

## Results

### Overview

This pilot study involved the development of a prototype of the CONTAV and MOVITA systems in a laboratory environment at the Integrated Systems Laboratory of the Interdisciplinary Center for Interactive Technologies, located in the Electronic Systems Engineering Department of the School Polytechnic of the University of São Paulo.

An interdisciplinary team composed of an occupational therapist and electrical and mechanical engineers specializing in interactive technologies aimed at health was essential to understand and address all aspects of offering a technological solution to real users.

A laboratory experimentation study was first conducted to develop the CONTAV prototype, which is a useful engineering research method to propose new techniques and investigate properties for product development [19]. In this technical evaluation of non–end user components, people with no disabilities and with adequate height and weight for the safe use of a motorized wheelchair with CONTAV hardware participated in testing the electronic home monitoring system hardware for positioning in the wheelchair.

To develop the MOVITA prototype, an exploratory study was conducted with an analysis of questionnaires from real users (eg, therapists and wheelchair users) based on the UCD methodology to propose solutions that consider real-world problems. This methodology allows the realization of coparticipatory research between developers and end users. In addition, many MOVITA functions were only inserted because of the survey of participants, such as the importance of inserting clinical guidelines in the app and having a screen to monitor the performance of functions to favor feedback.

Data analysis and the development of MOVITA’s functions, with the constant participation of end users in all stages of the project and the formative evaluation of the prototype via Figma, favored the acceptance of the system.

The prototype started with previous related works and, mainly, from the results of the systematic review by Gradim et al [16], which sought to identify works that presented ≥1 items of interest related to the “use of IoT systems in application in rehabilitation services” and addressed the development, architecture, application, implementation, use of sensors, and technological equipment in general, with evaluation of systems aimed at IoT in rehabilitation in the health area.

The use of cloud technology (IoT concept) with the possibility of data storage and an interoperable system were favorable points in the system and were praised by the participants, as well as the function screens for tilt and recline prescription in wheelchairs with typing fields for angles, duration, and frequency, as these factors are rarely addressed in the therapeutic service for wheelchair users.

The use of sensors from the IMU in an IoT solution was essential to provide alternatives to carry out the feedback of the actual use of the tilt and recline functions in the wheelchair, in an autonomous and programmable manner via a smartphone app, as well as a need raised by real users.

The components and concepts developed in this study and the formative assessment are presented in the next 3 sections: An Overview of the Prototype Architecture, IoT-Based System Architecture, and Formative Evaluation of Usability and Satisfaction.

### Overview of the Prototype Architecture

The architecture of the developed system aims to provide a technological solution with a central unit embedded in a wheelchair. It is an intelligent system composed of a layer of IMU sensors that are responsible for continuously acquiring patient data. This layer of sensors is directly connected to the central unit of the system, which is composed of an embedded computer capable of processing the data acquired from the patient sent to a smartphone using Bluetooth, which organizes the data and sends it to the network, which the therapist can access. The embedded computer also controls wheelchair actuators responsible for adjusting seat-back positions, such as tilt, recline, and footrest.

In addition, the system provides an interactive user interface to establish intuitive communication between the professional and patient. Finally, this system operates through an internet connection, enabling treatment data to be stored in a database accessible to health care professionals. They can use this database to monitor treatments and offer feedback to patients.

**Figure 1:**
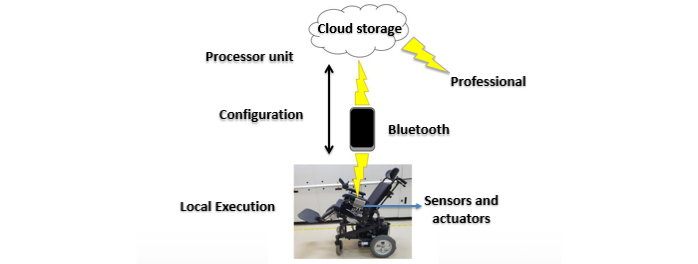
Architecture developed for the telemonitoring system of motorized wheelchair positions.

Figure 1 shows the overall system architecture (ie, IoT base, data acquisition, storage, and wheelchair-patient-clinician communication) for the power tilt and recline wheelchair monitoring system.

Initially, as shown in Figure 1, a set of sensors and actuators is embedded in the wheelchair, which must be adapted. These sensors are used in the wheelchair’s tilt, recline, and footrest functions and transmit data in a wireless operating mode. Subsequently, the data are stored and transmitted to the smartphone, which acts as an interface for sending the data to the cloud. The onboard system identifies the horizontal position of the seat regardless of the ground level and allows the user to autonomously prescribe positions for the user to perform in the wheelchair. The internet connection is only required for configuration, that is, it is not mandatory to use the internet connection during operation.

The proposed system architecture aims to benefit from IoT advantages, particularly for home-based rehabilitation monitoring. Telemonitoring, following the methodological model proposed by Bisio et al [20], emphasizes the structuring of rehabilitation in 4 aspects:

1. Users (rehabilitation patients)
2. Technological equipment, such as sensors, devices, and systems for measuring movement and activities
3. Hubs (function of connecting computers in a network, allowing telecommunications for the distribution of information among connected computers to a final destination)
4. Professionals and health units.

By using methods for classifying embedded technologies and sensors and connecting to the internet network, the system may be able to collect and transmit data from users to professionals and health care units. With this electronic system, CONTAV, it is possible to collect data on postural changes in the wheelchair (eg, variations of angles, frequency, and duration of positioning functions) daily to support the assessment and therapeutic prescription by the professional for the patient.

The structure of this system aims to compact the collected data and upload them to a cloud server, which, with a set-up application, can offer the analyzed data for feedback to the therapist and wheelchair users.

### IoT-Based System Architecture

**Figure 2:**
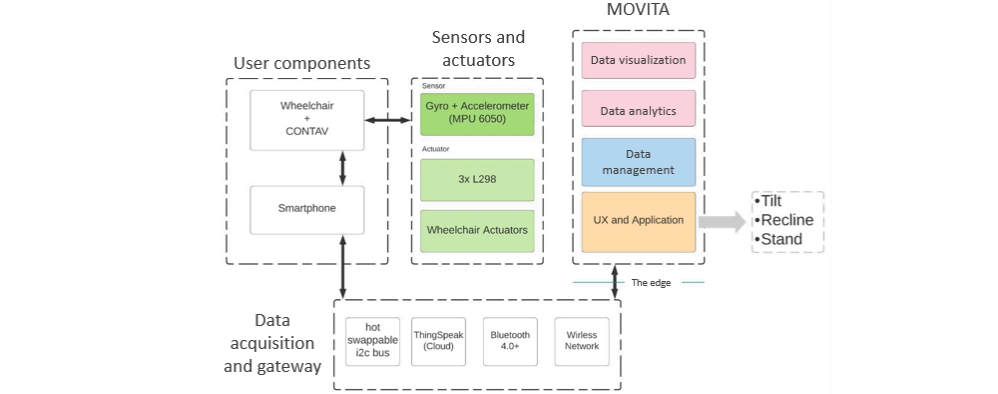
The system architecture.

The system architecture contains basic elements that comprise IoT applications for telemonitoring in rehabilitation: the user, professional, multisensory, and person and object connectivity. To develop and implement this system and manage home rehabilitation, the use of applicable and intelligent technologies with a multisensory framework, such as IoT, is recommended (Figure 2). The IoT provides an opportunity to implement the planned platform, with a caveat of adapting the technology to the proposals of health care services [8].

MOVITA describes the interface between the hardware and the user to simplify how rehabilitation professionals can use CONTAV to gather feedback. First, sensors and actuators were embedded to gather data from users based on preconfigured exercises. The collected data were sent to smartphones (using Bluetooth) and ThingSpeak (MathWorks; open source software allowing users to communicate with devices connected to the internet) using http and JSON patterns. Subsequently, a graphical user interface based on HTML 5 helps professionals to see or follow and interact with data sent by CONTAV+MOVITA.

We hypothesized that after gathering a large amount of data, we could improve the data visualization experience and start a new step: data science, intelligence of business, and prediction systems. Thus, MOVITA is also responsible for the data visualization layer and data management. Furthermore, we investigated strategies to show data and to improve user experience.

IoT characteristics have been implemented in which various tracking sensors can be used to collect data on the movements and activities of the wheelchair. Sensors provide information about positioning changes (with measurements of tilt and recline angles and their frequency), which can provide useful feedback for rehabilitation professionals.

In this context, 2 main tracking functions can be highlighted: movement within different environments (eg, social and home) and the recognition of a specific positioning activity. The first function aims to identify movements that the patient performs on a daily basis; at home; in a public, social environment, that is, collecting data on whether the patient is immobile or moving (wheelchair displacement). The second function aims to capture more specific movements related to physical rehabilitation, such as changing seat-back positions (tilt and recline) [8,20].

A study developed to design an electric wheelchair using Bluetooth [14] proposed a hardware architecture to establish reliable communication between different electronic parts, such as the power module, control module, and external devices using Bluetooth. The CONTAV project developed a technological solution in engineering capable of automating the process of tilting the seat (tilt and recline) of motorized wheelchairs with these functions.

From this project, financed by FINEP/Brazil, we developed hardware capable of controlling up to 3 motorized wheelchair actuators, inhibiting sensors for each seat function, and monitoring the seat angle with the MPU-6050 IMU sensor module, which has a 3-axis gyroscope and a 3-axis accelerometer on the same chip, and microelectromechanical systems technology.

**Figure 3:**
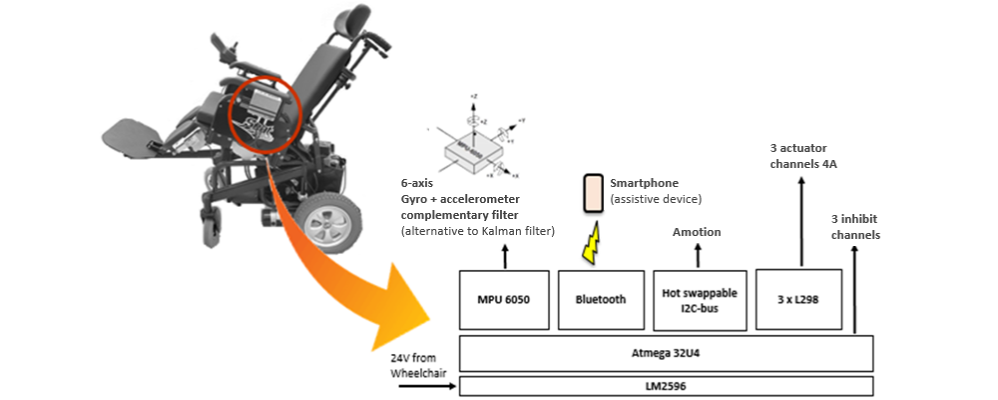
CONTAV (cadeira de rodas motorizada com CONTrole AVançado) hardware system developed for controlling the seat functions of a motorized wheelchair.

The CONTAV system can communicate via the I2C bus with the AMOTION motion control system (another group project), which is responsible for the power wheelchair movement. Figure 3 shows the CONTAV hardware system. The CONTAV system can also be stacked to double the number of treated actuators and the seating function.

CONTAV is an integrated system developed to increase specifications in a motorized wheelchair with intelligent functions and a wide range of inputs, in addition to the functions already existing in the trade.

The adaptations made at CONTAV for this research were based on the IoT technology. A wheelchair with this integrated hardware system can be expressed as follows:

1. Controlled by smartphone
2. Programmable and automatic tilt functions with the possibility of constant angle variations (frequency, angle, and duration)
3. Sensors identify the horizontal position of the seat, which serves as a reference, regardless of the level of the floor
4. Receives programming from the cloud via the web so that the health professional can define a base angle for tilt, angle for variations, and the period in which the variations should occur
5. Professional tracks (in the cloud via the web) record movements performed in a wheelchair (manually or autonomously)

**Figure 4:**
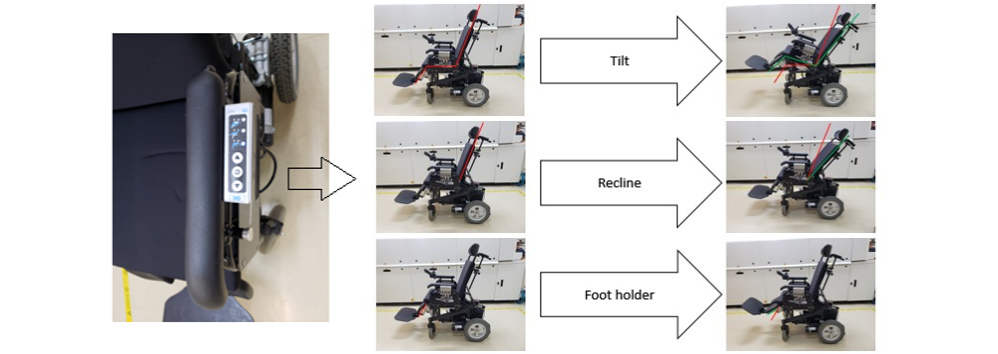
Tilt, recline, and foot holder (or footrest) on the motorized wheelchair controlled by smartphone (illustrated in CONTAV [cadeira de rodas motorizada com CONTrole AVançado]).

In a wheelchair, the CONTAV electronic system is responsible for controlling the actuators of the seat functions, such as tilt, recline, and foot support movement (Figure 4).

CONTAV is an original project developed using microelectronics and is attached to a wheelchair. CONTAV can control the actuators using the buttons on its panel, a joystick (when integrated with the AMOTION motion control system), and a mobile app. The wheelchair angle limits varied according to the device model and brand.

CONTAV has Bluetooth communication with a smartphone, which allows both instructions to be received and data to be sent. This allows the configuration of specific seat angles as well as the periodicity of the exchange between these angles. It also allows sending the registration information of these positions to the cloud so that the health professionals can analyze whether these angles and their variation in time are effective for the well-being of wheelchair users.

**Figure 5:**
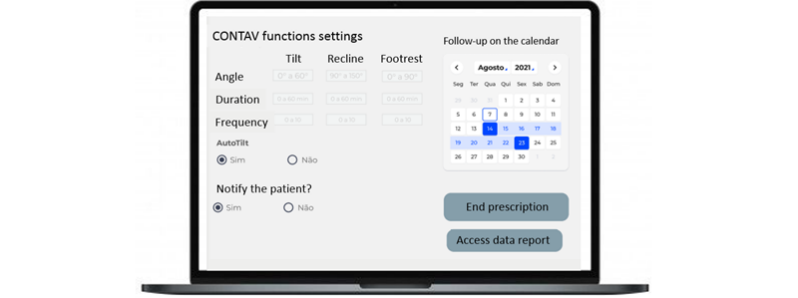
Prescription screen in the software using CONTAV (cadeira de rodas motorizada com CONTrole AVançado) functions.

CONTAV has a programmer to be used by the manufacturer to adjust the features to different power wheelchairs. Figure 5 shows a user interface view with the main CONTAV features. The therapist used this web-responsive front end to indicate the tilt base angle, angle variation, and automatic tilt periodicity. It also contains the option to end the prescription and access the data report.

The wheelchair user can disable an automatic tilt at any time for safety reasons.

**Figure 6:**
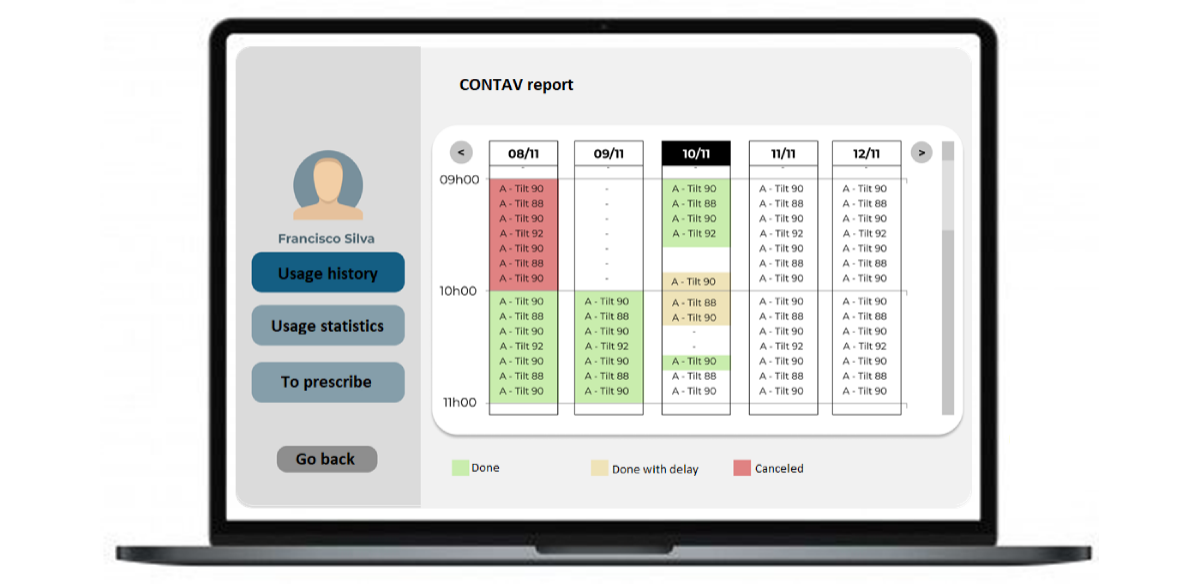
Wheelchair function use record screen.

The therapist could view the use log-in the web-responsive front end (Figure 6), by the MOVITA interface, showing the effective tilt angle variation during the days. This is very important for long-term follow-up, to understand the effectiveness of the indicated small angle variation in redistributing pressure in the buttocks and back area, and to prevent pressure injuries.

MOVITA is the development of a remote monitoring and follow-up platform based on the IoT for wheelchairs, open hardware, and software, with an interface between wheelchair users (patients and therapists), mobile devices, and a wheelchair (with a motorized system, sensors, and telemetry systems).

The web platform (single-page application and progressive web application) was initially prototyped by Figma, Inc (license: Figma terms of service), a web application to design a stand-alone mobile app or web application. This strategy allows us to collect user impressions in the early stages of development to ensure that a smooth process is assessed by the user using a co-design approach (which helps in designing a software solution that is feasible, viable, and desirable).

On the system’s web platform, the therapist has access to the patient’s information with reports of the data transmitted by the wheelchair and by the wheelchair user, allowing the therapist to create or change or delete prescriptions (being automated, in cases of wheelchair motorized or not) of treatments directly by the platform.

Furthermore, users primarily access the platform through their smartphones. They can receive prescription notifications and updates, regardless of their location. Users can also manage their prescriptions, especially the automated ones, and access reports detailing the progress of their treatment.

Tasks include therapeutic activities for motor and cognitive stimulation, prescription of wheelchair positions, indication of games (gaming therapy in rehabilitation), and exchange of messages between users with data sent (eg, texts, images, videos, audio, and documents). All these tasks (except the last one) can be planned by the health professional for use in person (in a therapeutic environment) or at a distance (in a home environment).

In addition, the system has a screen with guidance to health professionals regarding the specifications for prescribing variable positions in the wheelchair related to angles, duration, frequency, and order of actuators in the wheelchair, based on published scientific studies and documents or guidelines reference on this subject [9].

The planning of the tasks in the system includes the professional’s practice experience (professional expertise), the patient’s assessment, and therapeutic objectives, so that the tasks are adequate to what the therapist wants to achieve. Each patient may have an individualized and different prescription.

### Formative Evaluation of Usability and Satisfaction

The functionality buttons on each system screen were evaluated for each prototype presentation (UCD 1 and UCD 2). The applied evaluations were ASQ and SUS.

The ASQ scores decreased between sessions 1 (mean 1.13, SD 0.35) and 2 (mean 1.07, SD 0.26), demonstrating increased user satisfaction with the system in this research period.

After the first formative evaluation of the system with the application of the SUS and ASQ scales, we changed the 3 user views to improve user experience.

The results obtained from the SUS of each evaluator in sessions 1 (V1) and 2 (V2) are shown in Table 2.

Even with good results for usability and satisfaction, the team discussed all the results obtained in each session and the evaluators’ comments in subjective evaluations and, by consensus, made decisions about modifications to the system. Using this process iteratively, the design and functions were improved to meet user requirements.

**Table 2:** Total System Usability Scale score of each evaluator for a formative evaluation (n=5).

Evaluator	V1	V2
1	90	92.5
2	90	92.5
3	92.5	92.5
4	85	87.5
5	85	90

## Discussion

### Principal Findings

For the development of the entire system reported in this paper, three approaches to general architecture were considered for feedback: (1) user therapist, (2) user patient or client, and (3) IoT-based system. These strands were examined based on prior research applied to various technologies, the researchers’ expertise in the field, and their importance in enhancing the functionality and usability of an electronic health system [8,16]. An electronic system based on embedded technology (IoT) was inserted into the CONTAV to manually or automatically control the seating function of the power wheelchair.

A dynamic wheelchair system is prescribed to provide health, postural comfort, rest, and relief from pressure points. For effective and adequate use, guidelines should be based on an analysis of the application within the user’s context. However, none of the participants reported following these guidelines or professional monitoring.

Wheelchair users are already concerned about the seating posture because of the possibility of muscle atrophy, contractures, and worsening of the clinical and functional condition in general. In addition to the concerns arising from the use of wheelchairs, variations in positioning are an important factor for health conditions.

Lack of movement disfavors the dynamism of posture, which can aggravate health conditions. Therefore, therapeutic monitoring with a specialized professional for correct guidelines regarding angles and positions, personalized for each case, can assist in this process.

Literature indicates that tilting and reclining, combined with professional support and monitoring, are effective tools for the use of such systems to change positions with a tendency to reduce pressure. From this perspective, the MOVITA system with individualized prescriptions is viable, considering the patient, the different angles, frequency, and performing constant monitoring with faithful health indicators for real monitoring and feedback.

On the basis of the research experience of José and de Deus Lopes [13], CONTAV already has a smartphone app for wheelchair control and inputs for different devices for wheelchair control and functions. Having the option of autonomous control does not exclude manual control of the wheelchair by the user, as the wheelchair works normally, regardless of the CONTAV system. In other words, the user has the autonomy to choose whether to activate the system.

For this, the development of an electronic system with the ability to program via the web and the possibility of commands so that the clinic can prescribe the necessary positions, together with the period necessary for the user’s wheelchair to perform according to the proposal, help improve the performance of the therapeutic process.

The techniques for postural changes and relief of pressure points in a wheelchair are effective and necessary for the health of wheelchair users. Therefore, postural maneuvers should be one of the strategies used by users. To effectively contribute to the reduction of postural health complications such as contractures and pressure injuries, it is essential that users have access to professional information and necessary therapeutic monitoring. In addition, monitoring and access to information generate important economic impacts on the health system, quality of life, and health of users [21].

Maintaining the time of bone prominence on an external surface without movement causes the tissue in the gluteal region to harden, generating a stress mechanism in the region when the person remembers to move and causing an increase in deformities in the tissue; when local scars are already present, the regions are even more susceptible to damage [4].

Specifically, in the wheelchair seating position, the dynamic positioning system may promote a linear redistribution of pressure in the buttocks and back area (seat and backrest) when the angles are assessed and prescribed individually for each wheelchair user. However, some common factors can be highlighted regarding the use of tilt and reclining functions and angle orientation to prevent injuries. Stroupe et al [21] and Groah et al [22] reported that these 2 systems, as well as the angulations performed, must be prescribed and used properly. In this context, there is a need for future studies on the effects of both tilt and recline on the occupational engagement of wheelchair users approaching the interface of technology and human occupation.

Considering the viability of an electronic telemonitoring system, wheelchair users must maintain their autonomy and functionality. Therefore, this type of personalized electronic system emphasizes the importance of considering the environment, furniture, contexts, and routines of users with regard to their quality of life, health, and functionality.

The insertion of CONTAV and MOVITA in the provision of assessment and therapeutic follow-up services for wheelchair users tends to add evidence for specialized prescriptions and professional health follow-up (specialized and quality treatment) in addition to incorporating functions with a holistic view of the individual who uses such technology.

An evidence-based automated system with remote monitoring of wheelchair functions, based on IoT and IMU, embedded in a power wheelchair for telemonitoring positions with the user (therapist and wheelchair user) at the center of the functions, such as CONTAV and MOVITA, can help the clinician with real feedback for therapeutic decision-making and ongoing treatment [23]. In addition, this approach may provide relevant results for therapeutic treatment and other considerations regarding home care.

### Limitations

Although the methods and assumptions adopted in this study were preliminary, we believe that compared with simple tests, technical assessment from a causal perspective may be more appropriate for long-term observational data extracted from the real world rather than clinical trials. In addition, to assess the quality of management based on IoT, we proposed a simple evaluation method based on a typical clinical prescription.

The proposed components were part of the monitoring process measures from the perspective of using an electronic system. The results of the preliminary assessment were relatively objective and provided a quick understanding of the efficiency of telemonitoring functions.

A few potential weaknesses must be recognized in relation to this study.

Currently, there is no assessment of our system in actual practice with wheelchair users. The importance of evaluating our system on a large scale needs to be explored.

Although we have highlighted the characteristics of hardware and software for an electronic system in this study, it is important to emphasize that the technical specification is not the focus of assistive technology. For an effective assistive technology system, users’ daily life characteristics must be considered.

Specifically, in the case of the target audience being wheelchair users, it includes assessment of the seat and positioning, prescription and adaptation of the dynamic positioning system of the wheelchair (tilt and recline), comfort, context of usability of the wheelchair, occupational routine, and functional skills of the wheelchair.

Although tilt and recline can contribute to changes in posture and pressure relief, these other variables are extremely important to consider [10]. These aspects were considered in the general design and are not presented in this study because of the specific objective of presenting the hardware components of the system.

### Conclusions

The technologies used in the proposed system, such as the use of IMU sensors in an IoT solution, were central to providing alternatives that were sought to perform the functions of tilting and reclining in a wheelchair in an autonomous and programmable manner via a smartphone app. These concepts can bring greater benefits to telemonitoring and favor real feedback for professionals, allowing greater quality in the provision of health services for wheelchair users.

Further studies are needed, such as on the influence of constant monitoring on rehabilitation of wheelchair users, users’ behavior regarding pressure injury prevention measures and remote therapeutic follow-up, the effects of postural changes in the trunk and spine on the distribution of pressure during occupational routines and in activities of daily living, and how tilt and recline affect the functionality of wheelchair users.

The results of this study are preliminary, with important discoveries of concepts for the development of hardware and software for an automated electronic system in a motorized wheelchair for telemonitoring. We emphasize the importance of future studies on the correlation between different diagnoses and the use of the system in a real environment to help professionals prevent pressure injuries.

The next steps focus on user experience improvement and the development of new features using a co-design approach. However, the focus was on the user’s wheelchair in a different Brazilian context. More elements related to the treatment continuum (eg, protocols, assessment, and technologies associated with the intervention) can be incorporated into the system to improve therapy compliance with feedback to the professional and self-management of the user.

We also plan to implement our system in a wheelchair user community and assess the system on a larger scale.

In future work, the necessary evaluations and user-centered approaches for the development of an electronic monitoring system will be addressed, focusing on the positioning of motorized wheelchair users and possible partnerships with stakeholders.

## Abbreviations

ASQ: After-Scenario Questionnaire
CONTAV: cadeira de rodas motorizada com CONTrole AVançado
IMU: inertial measurement unit
IoT: Internet of Things
ISO: International Organization for Standardization
SUS: System Usability Scale
UCD: user-centered design
